# Gas Chromatography–Mass Spectrometry Reveals Stage-Specific Metabolic Signatures of Ankylosing Spondylitis

**DOI:** 10.3390/metabo13101058

**Published:** 2023-10-07

**Authors:** Yixuan Guo, Shuangshuang Wei, Mengdi Yin, Dandan Cao, Yiling Li, Chengping Wen, Jia Zhou

**Affiliations:** 1 Institute of Basic Research in Clinical Medicine, College of Basic Medical Sciences, Zhejiang Chinese Medical University, Hangzhou 310053, China; guoyixuan1725@gmail.com (Y.G.); w91315@gmail.com (S.W.); 202111114811060@zcmu.edu.cn (M.Y.); 202211110211002@zcmu.edu.cn (D.C.); 202211110211021@zcmu.edu.cn (Y.L.); 2 Key Laboratory of Chinese Medicine Rheumatology of Zhejiang Province, Zhejiang Chinese Medical University, Hangzhou 310053, China

**Keywords:** ankylosing spondylitis, metabolomics, gas chromatography–mass spectrometry, acute and remission stages

## Abstract

Ankylosing spondylitis (AS) is a type of chronic rheumatic immune disease, and the crucial point of AS treatment is identifying the correct stage of the disease. However, there is a lack of effective diagnostic methods for AS staging. The primary objective of this study was to perform an untargeted metabolomic approach in AS patients in an effort to reveal metabolic differences between patients in remission and acute stages. Serum samples from 40 controls and 57 AS patients were analyzed via gas chromatography–mass spectrometry (GC–MS). Twenty-four kinds of differential metabolites were identified between the healthy controls and AS patients, mainly involving valine/leucine/isoleucine biosynthesis and degradation, phenylalanine/tyrosine/tryptophan biosynthesis, glutathione metabolism, etc. Furthermore, the levels of fatty acids (linoleate, dodecanoate, hexadecanoate, and octadecanoate), amino acids (serine and pyroglutamate), 2-hydroxybutanoate, glucose, etc., were lower in patients in the acute stage than those in the remission stage, which may be associated with the aggravated inflammatory response and elevated oxidative stress in the acute stage. Multiple stage-specific metabolites were significantly correlated with inflammatory indicators (CRP and ESR). In addition, the combination of serum 2-hydroxybutanoate and hexadecanoate plays a significant role in the diagnosis of AS stages. These metabolomics-based findings provide new perspectives for AS staging, treatment, and pathogenesis studies.

## 1. Introduction

Ankylosing spondylitis (AS) is a systemic disease dominated by chronic inflammation of the axial joints, which mainly involve the sacroiliac joint, spine bone protrusion, paraspinal soft tissue, and peripheral joints, and it is often accompanied by varying degrees of extra-articular manifestations including ocular and gastrointestinal systems [1,2]. The pathogenesis of ankylosing spondylitis involves abnormalities of the immune system and chronic inflammation, and AS has a genetic predisposition with a strong association with the HLA–B27 gene [3,4].

AS is a chronic, incurable disease called undead cancer. Its clinical treatment focuses on suppressing inflammation, improving symptoms, preventing bone destruction, and decelerating disease progression [5]. Particularly, AS patients in acute and remission stages require different treatment strategies. During the acute stage, pain and inflammation may worsen, and non-steroidal anti-inflammatory drugs (NSAIDs) can help relieve pain and reduce inflammation [6,7]. Adequate rest is also necessary to prevent further joint damage. The remission stage, on the other hand, focuses on maintaining joint mobility and stability. Through appropriate exercises and treatments, stiffness and deformities of the joints can be slowed, and the risk of further complications can be reduced. Therefore, accurate diagnosis and prediction of the acute and remission stages of AS contributes to the development of personalized treatment plans, thereby effectively slowing the progression of the disease, improving clinical symptoms, and enhancing the quality of life for patients. Presently, the judgement of AS stage primarily relies on clinical manifestations (morning stiffness, pain, inflammation, etc.) and imaging examinations (X-rays, MRI, or CT) [8,9]. The erythrocyte sedimentation rate (ESR) and C-reactive protein (CRP) levels are important in the evaluation of disease progression to some extent, but these inflammatory indicators are currently only used as a reference due to their low specificity [10].

Metabolomics is a simultaneous qualitative and quantitative analysis of all metabolites in a specific biological sample under defined conditions, aiming to gain insight into the dynamics of metabolic networks in organisms, and thus, to reveal changes in disease progression and physiological states [11,12]. Metabolomics has gained significant attention in the field of disease research as it provides valuable insights into the metabolic alterations associated with the disease [13,14,15]. Targeted and untargeted metabolomics and lipidomics provide strong support for the diagnosis, prognosis, and treatment of gout, diabetes, SLE, cancer, etc. [16,17,18,19]. Several metabolomics studies have been conducted to investigate the metabolic profile of AS patients utilizing different analytical techniques such as mass spectrometry and nuclear magnetic resonance spectroscopy, aiming to identify potential biomarkers and unravel the underlying metabolic pathways involved in AS [20,21]. Perturbations in various metabolites such as tryptophan, lysine, proline, serine, and alanine have been discovered in AS patients [22,23,24,25,26,27], which provides information for exploring the pathogenesis of AS.

The pathological states of patients are different at various disease stages, and these differences can be manifested through alterations in metabolite composition and metabolic pathways. Therefore, investigating shifts in the humoral metabolome can help characterize the disease stages. However, metabolic changes associated with different stages of AS patients have not yet been investigated. The primary objective of this study was to perform a GC–MS-based untargeted metabolomic strategy in AS patients in an effort to identify metabolic differences between patients in remission and acute stages. Discriminant models were constructed to help differentiate AS patients at different stages based on the acquired metabolic data. Furthermore, we sought to reveal potential metabolic biomarkers to enable a more precise discrimination of AS stages. The findings may be useful in the clinical management of AS by providing valuable information for the diagnosis, treatment, and monitoring of patients.

## 2. Materials and Methods

### 2.1. Study Participants

A total of 57 patients (including 17 patients in the acute stage) in accordance with the New York Criteria for AS revised by the American College of Rheumatology in 1984 were included in the study. Meanwhile, an age- and gender-matched healthy control population of 40 cases was also enrolled. Subjects with severe cardiovascular, hepatic, and other organic pathologies and psychiatric abnormalities, as well as pregnant or lactating women were excluded. The study protocol was approved by the Ethics Committee of the First Affiliated Hospital of Zhejiang Chinese Medical University (2021-KLJ-010-03), and all patients signed an informed consent form.

### 2.2. Sample Preparation and Metabolomics Analysis

Fasting elbow venous blood of each subject was collected in the morning, followed by centrifugation, and the upper serum was stored at −80 °C for further testing. Before analysis, serum samples were thawed and vortex-mixed, and an aliquot of 50 μL of serum was spiked with 200 μL of acetonitrile (all operations were carried out on ice). After vortex-mixing, the samples were centrifuged at 4 °C for 10 min at 12,000 g to remove precipitated proteins, and the supernatant were freeze-dried under vacuum. Afterward, freeze-dried extracts were dissolved in 50 μL of methoxyamine pyridine solution (20 mg/mL), and the reaction mixtures were heated in a water bath at 40 °C for 90 min; then, 50 μL of MSTFA (N-trimethylsilyl-N-methyl trifluoroacetamide) was added to the samples for silylation reaction, followed by heating at 40 °C for 60 min. All samples to be analyzed were mixed in equal volumes to create quality control (QC) samples, and QC samples were prepared according to the aforementioned protocol.

All samples were analyzed on an Agilent 7890A gas chromatograph coupled with a 5975C MSD system (Agilent Technologies Inc., Santa Clara, CA, USA), and DB–5MS capillary column (30 m × 0.25 mm × 0.25 μm) was applied for separation. The carrier gas was helium (99.9996%) at a flow rate of 1.2 mL/min. The initial column temperature was kept at 70 °C for 3 min, programmed to 300 °C at a rate of 5 °C/min, and kept constant at 300 °C for 5 min. The injection volume was 1 μL, and the split ratio was 5:1. The injection port temperature and the transfer line temperature were set to 300 °C and 280 °C, respectively.

Mass spectrometry parameters were as follows: the ion source temperature was 230 °C, the scan range was 33~600 amu, and the solvent delay was 4.8 min. All samples were analyzed in a random order and QC samples were analyzed at every seven test samples to monitor the instrument stability.

### 2.3. Statistical Analysis

The QC sample is a homogeneous mix of all samples to be tested and contains the majority of the metabolite information in samples, so it was used as a template to establish a quantitative table of metabolites. The metabolic profile of the QC sample was firstly subjected to peak identification and overlapping peak resolution using AMDIS 2.62 (NIST, Boulder, CO, USA) software to obtain a quantitative table containing retention time and mass-to-charge ratio information of each metabolite. Then, the original data of all samples were imported into the workstation to integrate according to the above established table, and the peak area of each metabolite was obtained and combined into a two-dimensional data matrix.

After peak area normalization, data from all subjects were subjected to unit variance (UV) scaling and principal component analysis (PCA) using SIMCA-P 14.0 (Umetrics AB, Umea, Sweden) to obtain an overview of the differences in serum metabolic phenotypes between AS patients and healthy controls. The data were then filtered via orthogonal signal correction (OSC) to remove variables irrelevant to classification, and partial least squares discriminant analysis (PLS–DA) was utilized to discover specific metabolic patterns in AS patients, further differentiating between AS patients in acute and remission stages. Permutation test (200 permutations) and CV–ANOVA were performed to check the validity of OSC PLS–DA models. VIP (variable importance on projection) is an important indicator used to screen for differential metabolites in multivariate analyses, and variables with large VIP values contribute to model classification to a greater extent. The threshold of VIP values should be selected and adjusted according to the research requirements and data characteristics. In general, a VIP value greater than 1 is a standard threshold commonly used in the field to identify the most important variables in PLS–DA models. SPSS 21.0 (International Business Machines Corp., Armonk, NY, USA) was employed to conduct *t*-test and ANOVA. Here, serum metabolites with VIP > 1.0 in OSC PLS–DA and significance test *p* value < 0.05 between AS patients and controls or patients in different AS stages were screened as important candidate metabolites pending further structural annotation. Identification of metabolites was achieved by matching the mass spectra with commercial mass spectral libraries using NIST MS Search 2.0 (National Institute of Standards and Technology, Gaithersburg, MD, USA). The candidate results with similarity degree higher than 80% were further verified with standard compounds. The identified metabolites were introduced into MetaboAnalyst 4.0 (https://www.metaboanalyst.ca) for enrichment analysis to investigate which pathways were disturbed in AS patients [28].

A diagnostic model for discriminating acute and remission AS stages was established through binary logistic regression analysis SPSS 21.0 (International Business Machines Corp., Armonk, NY, USA) based on the AS stage-specific metabolites, and the diagnostic efficacy of each metabolite or different metabolites combination were evaluated by drawing receiver operating characteristic (ROC) curves. Spearman correlation analysis was performed to evaluate the correlation of serum differential metabolites with ESR and CRP in patients in acute and remission stages.

## 3. Results

### 3.1. Characterization of the Subjects

The demographic and clinical information of the selected subjects is shown in Table 1. The AS patient group and the control group were compatible in terms of age, gender, and BMI, without significant differences; the levels of CRP, ESR, and WBC (white blood cells) in the AS group were higher than those in the control group, which was consistent with the clinical characteristics of AS. In addition, HLA–B27 was positive in 78.90% of AS patients.

### 3.2. Characteristics of the Serum Metabolomics Analysis

In this study, the typical total ion current (TIC) chromatograms of AS patients and healthy controls obtained via GC–MS are shown in Figure S1A. A total of 219 metabolites were identified, integrated, and statistically analyzed. In order to examine whether the analytical method was stable and reproducible, one QC was added to every seven samples in the analytical sequence for a total of fifteen QC samples. The relative standard deviation (RSD) of the peak area of each metabolite in the QCs was calculated. It was found that 191 metabolites had RSD less than 30%, and the cumulative peak area was 98.3% of the total peak area (Figure S1B). Moreover, all QC samples were found to be within a range of 2-fold SD based on the PCA analysis in Figure S1C. The results demonstrated that the sample pretreatment and instrumental analysis processes had sufficient stability and reproducibility, and the acquired metabolomics data were reliable.

### 3.3. Serum Metabolic Differences between AS Patients and Controls

**Figure 1:**
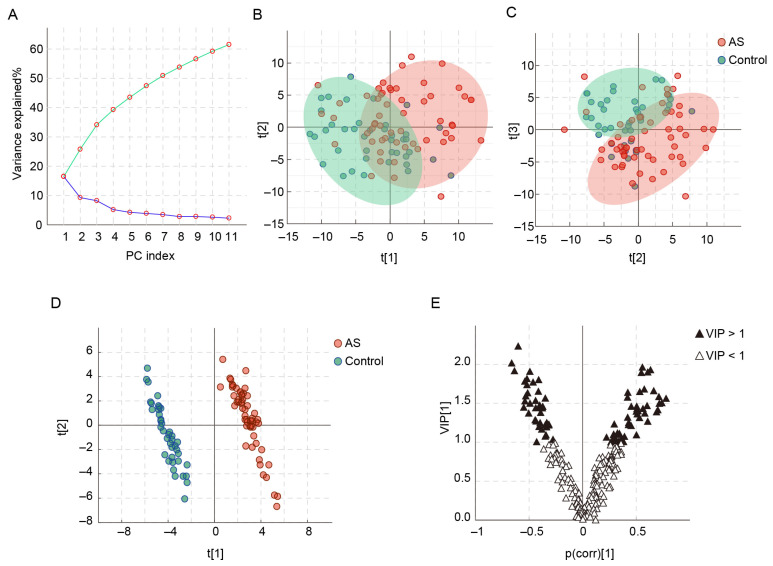
PCA and OSC PLS–DA of AS patients and healthy controls based on serum metabolic profiles. (**A**) PCA scree plot, (**B**) PCA score plot of PC1 and PC2, (**C**) PCA score plot of PC2 and PC3, (**D**) OSC PLS–DA score plot, and (**E**) V plot.

In the PCA analysis, the contribution of each principal component (PC) is shown in the scree plot (Figure 1A), which shows that approximately 61.5% of the variance in the raw data is explained by 11 PCs. As shown in the score plots (Figure 1B,C), there is a tendency for separation between AS patients and healthy controls, although there is some overlap. To identify the differences in metabolic profiles between AS patients and controls, OSC PLS–DA was carried out (Figure 1D). R2Y and Q2Y are used to evaluate the fitting effect of the OSC PLS–DA model. Here, both parameters are close to 1 (R2Y = 0.987, Q2Y = 0.965), which indicates that the model has good classification and prediction ability and can clearly distinguish between the two groups (Figure 1D). In the permutation test, all Q2 values are lower than the original points on the right, the intercept of the regression line at Q2 is less than zero, and all R2 values are lower than the original points on the right (Figure S2A). Moreover, the *p* value of the CV–ANOVA is less than 0.001. The above results indicate that the OSC PLS–DA model is not overfitted. The V plot presenting the VIP values and correlation coefficients of OSC PLS–DA model is shown in Figure 1E. The metabolites with VIP > 1.0 are located at both ends of “V”, where metabolites on the right side of the y-axis are positively correlated with AS patients and metabolites on the left side of the y-axis are negatively correlated with AS patients.

**Figure 2:**
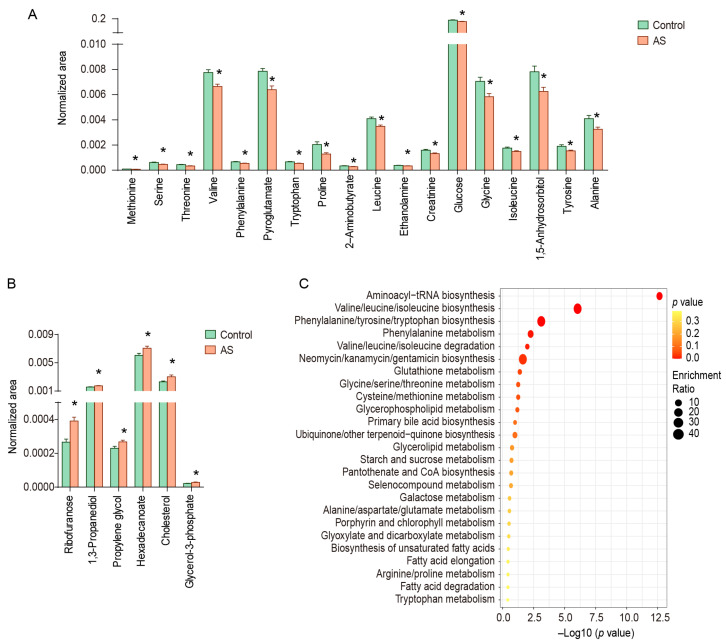
Differential metabolites and enrichment pathways in serum of AS patients and healthy controls. (**A**) Down-regulated metabolites in serum of AS patients, (**B**) up-regulated metabolites in serum of AS patients, (**C**) enriched pathways. * represents *p* < 0.05 between controls and AS patients.

The metabolites with VIP > 1.0 and *p* < 0.05 were considered to be metabolic characteristics of AS patients, and the structures of 24 metabolites have been identified, including amino acids and their derivatives (methionine, serine, threonine, valine, phenylalanine, pyroglutamate, tryptophan, proline, leucine, glycine, isoleucine, tyrosine, alanine, 2-aminobutyrate, and ethanolamine), lipids and their derivatives (hexadecanoate and glycerol-3-phosphate), carbohydrates and their derivatives (glucose, ribofuranose, propylene glycol, 1,5-anhydrosorbitol, and 1,3-propanediol), creatinine, and cholesterol (Figure 2A,B). These metabolites were mainly enriched in valine/leucine/isoleucine biosynthesis and degradation (threonine, leucine, isoleucine, and valine), phenylalanine/tyrosine/tryptophan biosynthesis (phenylalanine, tryptophan, and tyrosine), glutathione metabolism (glycine, alanine, and pyroglutamate), and so on (Figure 2C).

### 3.4. Serum Metabolic Differences between Acute and Remission Stages of AS Patients

**Figure 3:**
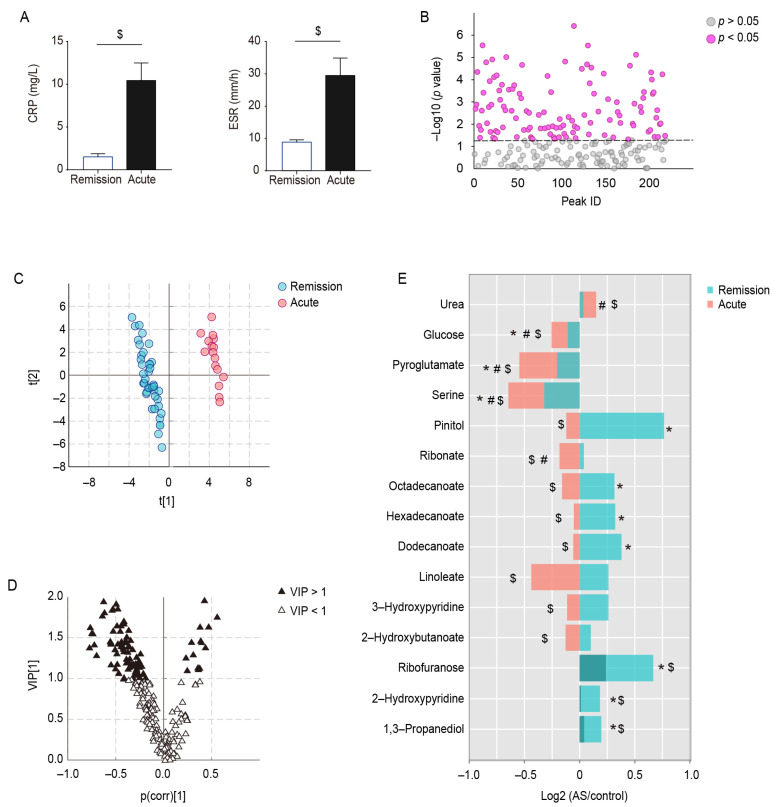
Metabolic differences between acute- and remission-stage patients with AS. (**A**) Differences in CRP and ESR in AS patients during different stages, (**B**) ANOVA of serum metabolites between controls, AS patients in the remission stage, and AS patients in the acute stage. (**C**) OSC PLS–DA score plot of AS patients in remission versus acute stages, (**D**) OSC PLS–DA V plot. (**E**) Trends of serum metabolites in AS patients during acute and remission stages. * represents *p* < 0.05 between controls and AS patients in the remission stage, # represents *p* < 0.05 between controls and AS patients in the acute stage, and $ represents *p* < 0.05 between AS patients in acute and remission stages.

Based on the clinical symptoms and inflammatory indicators of AS, the AS patients were differentiated into patients in the acute stage (17 cases) and patients in the remission stage (40 cases); there was no statistically significant difference between the two groups in terms of age, gender, and BMI, and the CRP and ESR indices of the patients in the acute stage were significantly higher than those of the patients in the remission stage (Figure 3A). Differences in metabolite levels between controls and acute- and remission-stage AS patients were compared using ANOVA (Figure 3B), and OSC PLS–DA analysis was also performed to distinguish between the different stages of AS patients (Figure 3C). The model parameters (R2Y = 0.985, Q2Y = 0.938) are close to 1, indicating that the OSC PLS–DA model fits the data well. The permutation test (Figure S2B) and CV–ANOVA (*p* < 0.001) support the goodness of fit of the model. Subsequently, the metabolites with VIP > 1.0 were marked in the OSC PLS–DA V plot, which may play a very important role in classification (Figure 3D).

Ultimately, 44 stage-specific metabolites were screened out on the basis of VIP and *p* value. Of these, 15/44 were identified, and the trends of these metabolites in the control group and patients during acute and remission stages are presented in Figure 3E. Serum levels of seven metabolites, 1,3-propanediol, 2-hydroxypyridine, ribofuranose, dodecanoate, hexadecanoate, octadecenoate, and pinitol, were higher in the AS patients than in controls during the remission stage, whereas they were reduced to control levels during the acute stage. Three metabolites (serine, pyroglutamate, and glucose) appeared to be reduced in AS patients during the remission stage compared with the controls, and the decrease was more pronounced during the acute stage. In addition, two metabolites were unchanged in AS patients during the remission stage and significantly changed during the acute stage (ribonate decreased and urea increased); and the levels of 2-hydroxybutanoate, 3-hydroxypyridine, and linoleate were significantly lower in the acute stage than in the remission stage.

**Figure 4:**
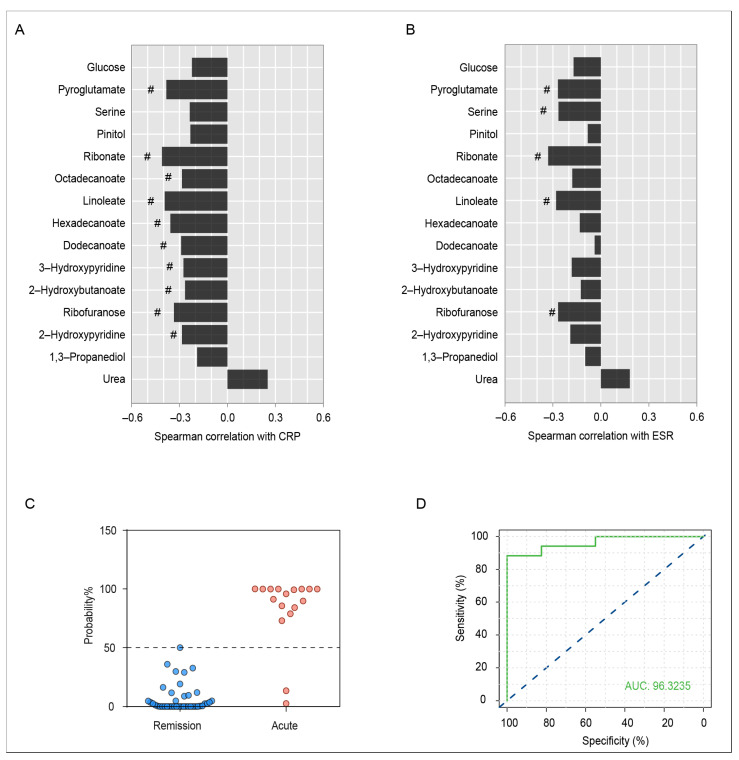
(**A**) Spearman correlation coefficient of serum differential metabolites and CRP index in AS patients during the remission and acute stages. # indicates a statistically significant correlation. (**B**) Spearman correlation coefficient of serum differential metabolites and ESR index in AS patients during the remission and acute stages. # indicates a statistically significant correlation. (**C**) Predictive probability of acute AS patients and remission patients based on serum differential metabolites. Individuals below the blue dashed line are predicted to be in remission stage, and those above the blue dashed line are predicted to be in the acute stage. (**D**) ROC analysis for the discrimination of AS patients during different stages using the combination of serum 2-hydroxybutanoate and hexadecanoate levels. The blue dashed line is the reference line for performance, and the ROC curve above the diagonal means the performance of the model is relatively good.

Spearman’s correlation analysis of serum differential metabolites with CRP and ESR in the acute and remission stages of AS patients was carried out (Figure 4A,B). There was a significant correlation between CRP levels and metabolites such as 2-hydroxypyridine, ribofuranose, 2-hydroxybutanoate, 3-hydroxypyridine, dodecanoate, hexadecanoate, linoleate, octadecanoate, ribonate, and pyroglutamate, and metabolites including ribofuranose, linoleate, ribonate, serine, and pyroglutamate were negatively correlated with ESR. Particularly, ribofuranose, linoleate, ribonate, and pyroglutamate had a strong negative correlation with both CRP and ESR, indicating that the changes in levels might be associated with the progression of AS.

Clinical manifestations, imaging examinations, and inflammatory indexes are important indicators of AS progression to some extent, but with low sensitivity and specificity for AS staging. In this study, some significantly different metabolites in the serum of AS patients during the acute and remission stages were discovered, which may have potential for the staging of AS. Through the binary logistic regression analysis, a model was established to distinguish the acute stage and remission stage of AS patients by the serum levels of 2-hydroxybutanoate and hexadecanoate. The model can effectively distinguish the different stages of AS (Figure 4C). ROC curve analysis showed that the combined application of serum 2-hydroxybutanoate and hexadecanoate had a good diagnostic effect for AS staging (AUC = 0.963, Figure 4D).

## 4. Discussion

AS is a chronic inflammatory systemic disease with still unknown etiology, and recent studies have recognized that it might be correlated with infection, autoimmune, and genetic factors [2,6,29]. Correct staging is very important for the treatment of AS. In the acute stage, the main goal is to relieve pain and inflammation in patients with AS as soon as possible, while in the remission stage, the focus is on maintaining joint mobility and stability and slowing the progression of the disease and the likelihood of flare-ups. However, there is a lack of effective diagnostic methods for staging AS. In this study, we found that valine/leucine/isoleucine biosynthesis and degradation (threonine, leucine, isoleucine, and valine), phenylalanine/tyrosine/tryptophan biosynthesis (phenylalanine, tryptophan, and tyrosine), and glutathione metabolism (glycine, alanine, and pyroglutamate) were altered in AS patients compared with healthy individuals. Furthermore, 15 metabolites showed a significant difference between AS patients in the acute and remission stages.

A large number of inflammatory factors can be generated during the course of AS. Some studies have reported that high levels of serum-free fatty acids could induce chronic low-grade inflammation [30]; concurrently, inflammatory mediators can interfere with lipid metabolism as well [31,32]. In this study, we found that the levels of dodecanoate, hexadecanoate, and octadecanoate were elevated in the remission stage of AS patients, whereas a decrease in fatty acids was observed in AS patients in the acute stage, which could be attributed to the intensified energy demand stemming from a more robust inflammatory response during the acute stage, and thus more serum fatty acids entering the mitochondrial β-oxidation to provide the necessary energy [33,34]. Importantly, the serum levels of fatty acids, including linoleate, dodecanoate, hexadecanoate, and octadecanoate, exhibited significant correlations with the inflammatory marker CRP, suggesting a potential linkage between the development of AS and the alterations in fatty acids.

Glucose catabolism is a major source of energy [35], and glycerol-3-phosphate is mainly derived from glucose metabolism. Decreased serum glucose and 1,5-anhydrosorbitol, along with elevated glycerol-3-phosphate in AS patients, suggest an accelerated glucose metabolism, possibly linked to an increased energy demand due to chronic inflammation [36]. 2-Hydroxybutanoate can also enter the energy metabolism pathway and participate in the production of adenosine triphosphate [37]. Compared with the remission stage of AS, patients in the acute stage experience heightened inflammatory responses and greater energy requirements, leading to further consumption of glucose and 2-hydroxybutanoate to provide additional energy for the body.

Multiple amino acid metabolic pathways and related metabolites showed abnormalities in AS patients. The biosynthesis and degradation of valine/leucine/isoleucine primarily influence the levels of branched-chain amino acids (BCAAs) [38], such as leucine, isoleucine, and valine. BCAAs play an important role in the metabolism and immune regulation of the body [39]. Several studies have indicated an association between BCAA levels and the inflammatory state [40]. In inflammatory diseases or infections, BCAAs can be converted into energy in the liver, thereby maintaining the energy balance of the body [41] and leading to the consumption of BCAAs during the inflammatory state. Here, the altered BCAA levels in AS patients, characterized by significant reductions in valine, leucine, and isoleucine, suggest that AS patients are experiencing sustained energy depletion.

Lymphocyte abnormality is one of the most important factors in the development of AS [42]. Amino acids are the basic substances that constitute proteins, which are closely related to various aspects of the immune system, including the development of immune organs, the differentiation and proliferation of immune cells, the secretion of cytokines, the production of antibodies, and so on [43,44,45]. Disruptions in the biosynthesis of phenylalanine/tyrosine/tryptophan and decreased tryptophan were observed in AS patients. Previous studies have demonstrated that tryptophan depletion and the accumulation of tryptophan metabolites can impede T cell function, including suppressing T cell proliferation, impairing T cell survival, and promoting T cell apoptosis [46,47]. Threonine and methionine are major amino acids comprising immunoglobulins, and methionine has a protective effect on lymphocytes. The decrease in threonine and methionine may inhibit the synthesis and secretion of immunoglobulins, affecting humoral immunity [43,48]. The change in these amino acids is closely associated with the immune dysfunction in AS patients.

In AS patients, the glutathione metabolism was significantly changed. With the increased level of oxidative stress in AS patients, the demand for antioxidants, such as glutathione, increases to counteract the excessive production of reactive oxygen species (ROS) [49,50]. Amino acids like glycine and alanine play crucial roles in the synthesis of glutathione [51,52]. Our research has revealed a reduction in these amino acids in AS patients, which could be associated with an increased demand for glutathione synthesis.

Notably, serine and pyroglutamate were consistently decreased in the acute stage of AS compared with the remission stage. Serine is one of the building blocks of neutrophil serine proteases (NSPs), which play a significant role in inflammatory responses and immune reactions during acute infections [53,54]. Pyroglutamate is considered an endogenous antioxidant, which may help neutralize intracellular ROS, thus protecting cells from damage caused by oxidative stress [55,56]. The sustained reduction in serine and pyroglutamate during the acute stage of AS implies that AS is characterized by inflammatory activation and oxidative damage during the acute stage.

The main limitations of the study are as follows: first, only a portion of the metabolites associated with AS stages were structurally identified, and the unidentified metabolites may have important biological functions, which need to be further confirmed in combination with other analytical techniques; second, we did not set up an independent validation set to evaluate the diagnostic efficacy of the potential biomarkers in AS staging in clinical practice.

## 5. Conclusions

Taken together, a GC–MS-based metabolomics analysis was performed to shed light on significant changes in AS patients. In particular, it was demonstrated that patients in the acute stage and remission stage showed different metabolic characteristics, especially in terms of the changes in fatty acids (linoleate, dodecanoate, hexadecanoate, and octadecanoate), amino acids (serine and pyroglutamate), 2-hydroxybutanoate, glucose, etc. We propose that these alterations may be related to the aggravated inflammatory response and elevated oxidative stress in the acute stage. Moreover, serum 2-hydroxybutanoate and hexadecanoate had good efficacy for the stage division of AS. All the aforementioned findings could provide a basis for the staging treatment and pathogenesis studies of AS.

## Figures and Tables

**Table 1:** The clinical information of AS patients and healthy controls.

Characteristics	Control (*n* = 40)	AS (*n* = 57)	*p*
Male/Female,	20/20	28/29	0.932
Age, y	36.08 ± 1.53	39.37 ± 1.61	0.158
BMI	22.22 ± 0.46	22.68±0.53	0.534
CRP (mg/L)	1.90 ± 0.38	4.18 ± 0.85	0.036
ESR (mm/h)	9.32 ± 0.67	15.02 ± 2.08	0.032
WBC (10^9^/L)	5.90 ± 0.19	6.66 ± 0.20	0.010
HLA–B27 (+/−)	2/38	45/12	<0.001

AS, ankylosing spondylitis; BMI, body mass index; CRP, C-reactive protein; ESR, erythrocyte sedimentation rate; WBC, white blood cell; HLA–B27, hydrophile-lipophile balance-27. Data are shown as mean ± SEM.

## Data Availability

The data presented in this study are available on request from the corresponding author. The data are not publicly available due to ethical restrictions.

## Supplementary Materials

The following supporting information can be downloaded at: https://www.mdpi.com/article/10.3390/metabo13101058/s1, Figure S1: Analytical characteristics of the serum metabolic profiling method; Figure S2: Validation plots for OSC PLS–DA models.
