# Supplementary material for: Gas Chromatography–Mass Spectrometry Reveals Stage-Specific Metabolic Signatures of Ankylosing Spondylitis
Source: Metabolites. 2023 Oct 7;13(10):1058. doi: 10.3390/metabo13101058 (PMC10608640; doi:10.3390/metabo13101058)
Supplement: Supplementary file 1 [file metabolites-13-01058-s001.zip › metabolites-2587874-supplementary.pdf]

metabolites

Supplementary Materials

Gas Chromatography–Mass Spectrometry Reveals Stage-Specific Metabolic Signatures of Ankylosing Spondylitis

Yixuan Guo [1,†], Shuangshuang Wei [1,†], Mengdi Yin [1], Dandan Cao [1], Yiling Li [1], Chengping Wen [1,2,*] and Jia Zhou [1,2,*]

[1] Institute of Basic Research in Clinical Medicine, College of Basic Medical Sciences, Zhejiang Chinese Medical University, Hangzhou 310053, China; guoyixuan1725@gmail.com (Y.G.); w91315@gmail.com (S.W.); 202111114811060@zcmu.edu.cn (M.Y.); 202211110211002@zcmu.edu.cn (D.C.); 202211110211021@zcmu.edu.cn (Y.L.)

[2] Key Laboratory of Chinese Medicine Rheumatology of Zhejiang Province, Zhejiang Chinese Medical University, Hangzhou 310053, China

* Correspondence: wencp@zcmu.edu.cn (C.W.); zhoujia@zcmu.edu.cn (J.Z.)

† These authors contributed equally to this work.

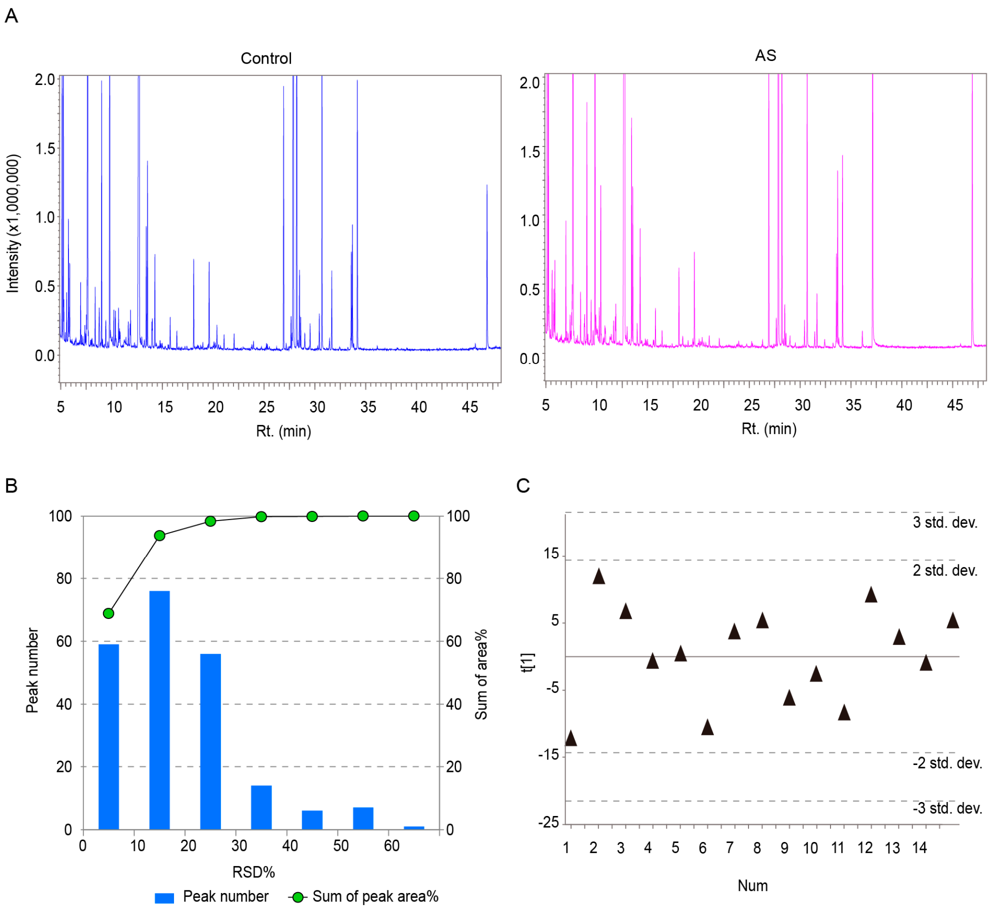

Figure S1. Analytical characteristics of the serum metabolic profiling method. (A) Representative TIC chromatograms of serum from patients with AS and healthy controls, (B) The distribution of the RSD% for metabolites in QCs, and (C) PCA score plot of QCs on PC1.

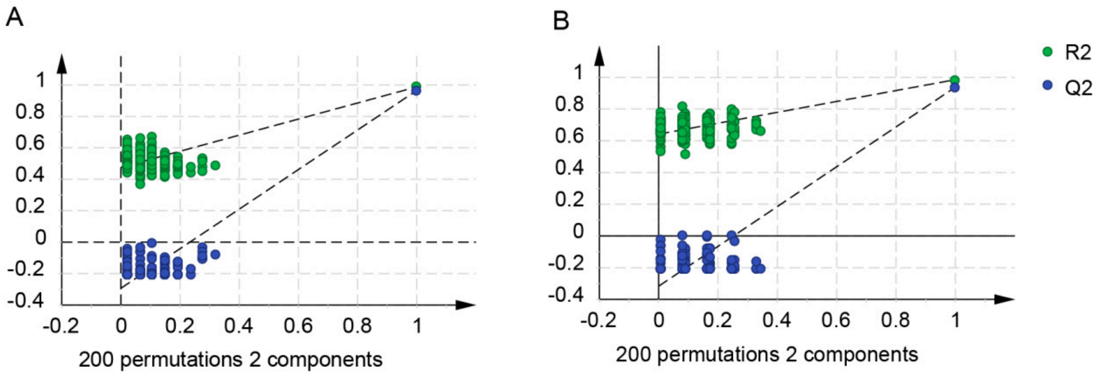

Figure S2. Validation plots for OSC PLS-DA models. (A) Controls vs AS patients, (B) remission stage vs acute stage.
